# Impact of type 2 diabetes mellitus on Alzheimer’s disease ‐related pathology preceding cognitive impairment

**DOI:** 10.1002/alz.092534

**Published:** 2025-01-09

**Authors:** Yang Yang Yu, Joseph Nowell, Paul Edison

**Affiliations:** ^1^ Imperial College London, London UK

## Abstract

**Background:**

Alzheimer’s disease and type 2 diabetes mellitus rank among the top ten leading global causes of death. The association between diabetes and Alzheimer’s is linked to chronic low‐grade inflammation, hyperinsulinemia, and the interplay between peripheral and central insulin resistance, influencing insulin signalling. We evaluated the association between diabetes and Alzheimer’s‐related neuropathology in cognitively unimpaired older adults with diabetes.

**Methods:**

We compared tau, cerebral glucose metabolism and amyloid in 147 cognitively normal participants, comprising 32 clinically diagnosed with diabetes and 115 without diabetes, from the Alzheimer’s Disease Neuroimaging Initiative dataset. We processed 329 available PET scans for ^18^F‐AV‐45, ^18^F‐flortaucipir and ^18^F‐fludeoxyglucose (FDG). Independent t‐tests and multivariate analysis were conducted for regions of interest (ROI) values, encompassing the global cortex and subsequent brain cortices, the medial temporal lobes, striatum, thalamus, and the anterior and posterior cingulate. Voxel‐wise analysis using SPM was also performed for these groups. Amyloid positive and negative subjects were evaluated separately.

**Results:**

Cognitively unimpaired diabetic subjects exhibited higher tau uptake compared to the non‐diabetic group in the whole brain (global brain value) and individual brain cortices, including the frontal, temporal, parietal, and occipital lobes. In diabetic subjects with negative amyloid status, significance was observed in the whole brain, as well as the parietal, temporal, medial temporal lobes, and the striatum. Reduced FDG uptake was only evident in diabetic subjects with positive amyloid status in the striatum. Increased amyloid deposition was noted in the medial temporal lobe and hippocampus in subjects with diabetes compared to those without.

**Conclusions:**

Neuropathological changes in the diabetic brain precede the manifestation of cognitive decline. Specifically, there is an increase in the presence of tau in the diabetic brain even in the absence of amyloid, indicating that tau may be the predominant driver of cognitive decline in diabetes.

